# Supplementary material for: Botulinum Neurotoxin A-Induced Muscle Morphology Changes in Children with Cerebral Palsy: A One-Year Follow-Up Study
Source: Toxins (Basel). 2025 Jun 27;17(7):327. doi: 10.3390/toxins17070327 (PMC12299369; doi:10.3390/toxins17070327)
Supplement: Supplementary file 1 [file toxins-17-00327-s001.zip › toxins-3590323-supplementary.pdf]

Botulinum Neurotoxin A-Induced Muscle Morphology Changes in Children with Cerebral Palsy: A One-Year Follow-Up Study

Charlotte **Lambrechts,** Nathalie **De Beukelaer ,** Ines **Vandekerckhove,** Ineke **Verreydt,** Anke **Andries,** Francesco **Cenni,** Ghislaine **Gayan-Ramirez,** Kaat **Desloovere and** Anja **Van Campenhout**

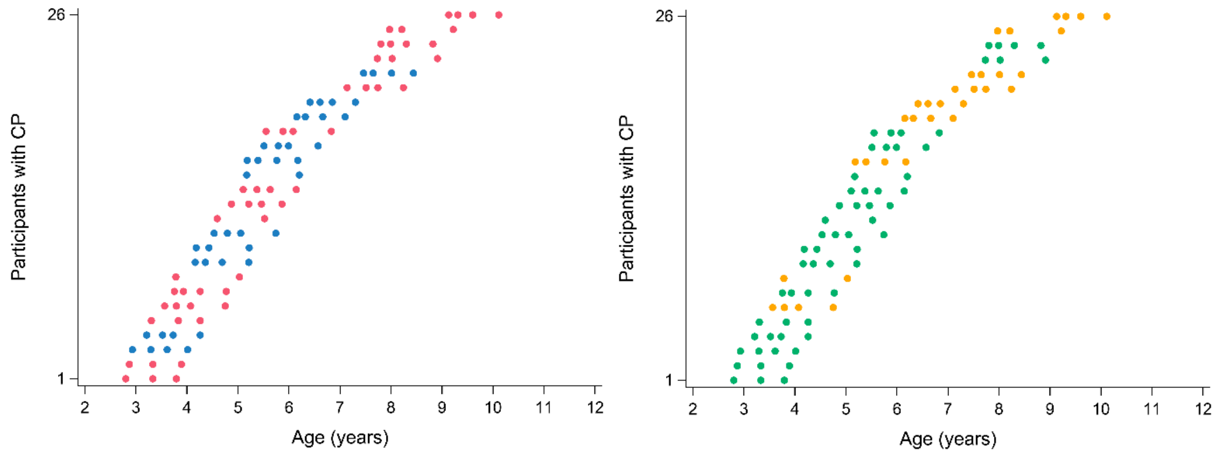

Figure S1. Visual outline of number of repeated assessments and time interval per participant across the age range. On the left graph, the blue dots indicate children with a Gross Motor Function Classification System (GMFCS) level I, and the red dots indicate children with a GMFCS level II+III. On the right graph, the green dots indicate children with a no history of previous BoNT-A injections, and the orange dots indicate children with a history of previous BoNT-A injections. CP, cerebral palsy.

Table S1. Overview of the baseline patient characteristics and muscle parameters of the intervention group at 4 different timepoints

	BL	3M	6M	1Y
Section A. Anthropometric data				
Sample size	n=26	n=20	n=20	n=26
Gender	female n=10 /male n=16	female n=9 /male n=11	female n=9 /male n=11	female n=10 /male n=16
Age (years)	5.19 (3.26)	5.87 (1.77)	5.60 (1.82)	6.24 (1.81)
Body length (m)	1.05 (0.13)	1.10 (0.13)	1.08 (0.13)	1.12 (0.14)
Body weight (kg)	17.97 (5.70)	19.69 (6.55)	18.81 (6.39)	20.60 (6.59)
GMFCS level	I n=11/II n=7/III n= 8	I n=10/II n=5/III n=5	I n=9/II n=5 /III n=6	I n=11/II n=7/III n= 8
Topographical involvement	unilateral n=10<br>bilateral n=16	unilateral n=9<br>bilateral n=11	unilateral n=7<br>bilateral n=13	unilateral n=10<br>bilateral n=16
BoNT-A naive/history	naive n=17/history n=9	naive n=12/history n=8	naive n=13/history n=7	naive n=17/history n=9
Section B. Estimates with confidence intervals of the muscle (morphology) parameters				
	Estimate (CI)	Estimate (CI)	Estimate (CI)	Estimate (CI)

zMV	-1.61 (-2.00,-1.21)	-2.11 (-2.54,-1.67)	-2.03 (-2.44,-1.61)	-1.95 (-2.38,-1.51)
zCSA	-1.50 (-1.97,-1.030)	-2.08 (-2.62,-1.54)	-2.30 (-2.83,-1.77)	-1.65 (-2.19,-1.10)
zML	-1.09 (-1.46,-0.73)	-1.03 (-1.49,-0.56)	-1.08 (-1.51,-0.66)	-1.07 (-1.45,-0.69)
EI	165.61 (160.69,170.54)	174.90 (169.08,180.71)	164.85 (159.82,169.88)	169.75 (165.16,174.33)
nMV (ml/kg*m)	1.40 (1.29,1.52)	1.26 (1.13,1.40)	1.30 (1.17,1.43)	1.32 (1.19,1.46)
nCSA (mm^2/kg)	18.20 (16.89,19.51)	16.54 (15.13,17.96)	15.95 (14.60,17.30)	17.60 (16.13,19.08)
nML (mm/m)	116.78 (112.55,121.01)	117.76 (112.42,123.10)	117.86 (113.07,122.64)	118.49 (114.21,122.78)

Data are presented as mean (standard deviation) for anthropometry (Section A). Estimates with confidence intervals (CI, lower bound, upper bound) are presented for the muscle (morphology) parameters (Section B). BL, baseline; 3M, 3 months; 6M, 6 months; 1Y, 1 year; n, number; m, meter; kg, kilogram; GMFCS, Gross Motor Function Classification System; BoNT-A, botulinum neurotoxin type A, zMV, z-scores of muscle volume; zCSA, z-scores of cross-sectional area; zML, z-scores of muscle length; EI, echo-intensity; nMV, normalized muscle volume; ml, mililiter; nCSA, normalized cross-sectional area; mm^2, squared milimeter; nML, normalized muscle length; mm, milimeter

Table S2. Results of the linear mixed model analyses of the normalized muscle size parameters of the intervention group

	nMV (ml/kg*m)			nCSA (mm^2/kg)			nML (mm/m)		
Time effect	F-statistics	p-value	η^2p	F-statistics	p-value	η^2p	F-statistics	p-value	η^2p
	3.74	0.015	0.15	6.18	**0.001***	0.23	0.60	0.62	0.067
Differences	MD/%	p-value		MD/%	p-value		MD/%	p-value	
3 months – baseline	-0.14/-10%	**0.006**		-1.66/-9%	0.014		0.98/+1%	0.47	
6 months – baseline	-0.10/-7%	**0.007**		-2.25/-12%	**0.0001***		1.08/+1%	0.54	
1 year – baseline	-0.08/-6%	0.023		-0.60/-3%	0.26		1.71/+1%	0.27	
6 months – 3 months	0.04/+3%	0.45		-0.59/-4%	0.25		0.10/0%	0.95	
1 year – 3 months	0.06/+5%	0.26		1.06/+6%	0.079		0.73/+1%	0.64	
1 year – 6 months	0.02/+2%	0.40		1.65/+10%	**0.002***		0.63/+1%	0.49	

Overview of the time effect and pairwise mean differences between the 4 timepoints. For the pairwise mean differences, significant results at the 0.013 corrected alpha-level were indicated in bold. Asterisks (*) indicate significant results at the 0.002 corrected alpha-level for 6 pairwise comparisons. Mean differences of the estimates were calculated as: outcome at later timepoint – outcome at earlier timepoint. Positive mean difference (MD) values indicate an increase of the muscle outcome at the follow-up measurement. nMV, normalized muscle volume; ml, mililiter; kg, kilogram; m, meter; nCSA, normalized cross-sectional area; mm2, squared milimeter; nML, normalized muscle length; mm, milimeter; η^2p, partial eta squared; MD, mean difference

Table S3. Comparison between the baseline patient characteristics and muscle parameters of the Gross Motor Function Classification System (GMFCS) I group and GMFCS II+III group

	GMFCS I	GMFCS II+III	p-value
Age (years)	5.17; 1.99	4.87; 4.17	0.90
Body length (m)	17.20; 3.45	16.50; 9.80	0.55
Body weight (kg)	1.05; 0.11	1.00; 0.35	0.82
zMV	-1.31; 1.24	-1.84; 1.21	0.031
zCSA	-1.36; 1.65	-1.67; 1.85	1.00
zML	-0.04; 1.93	-1.39; 1.07	0.058
EI	162.74; 10.00	163.32; 17.10	0.48
nMV (ml/kg*m)	1.54; 0.36	1.32; 0.30	**0.009**
nCSA (mm^2/kg)	18.51; 4.77	17.29; 4.58	0.70

nML (mm/m)	126.42; 17.25	114.31; 11.10	0.024

Data are presented as median; IQR (interquartile range). Mann-Whitney U test with significant alpha level 0.05 was applied for anthropometric parameters and were indicated in bold. Bonferroni correction was applied for 4 muscle outcomes (alpha level 0.013) and significant results were indicated in bold. GMFCS, Gross Motor Function Classification System, m, meter; kg, kilogram; zMV, z-scores of muscle volume; zCSA, z-scores of cross-sectional area; zML, z-scores of muscle length; EI, echo-intensity; nMV, normalized muscle volume; ml, mililiter; nCSA, normalized cross-sectional area; mm^2, squared milimeter; nML, normalized muscle length; mm, milimeter

Table S4. Overview of the muscle parameters at the 4 different timepoints per GMFCS group

	BL	3M	6M	1Y
	Estimate (CI)	Estimate (CI)	Estimate (CI)	Estimate (CI)
GMFCS I (n=11)				
zMV	-1.11 (-1.72,-0.51)	-1.73 (-2.35,-1.11)	-1.39 (-1.95,-0.82)	-1.23 (-1.77,-0.68)
zCSA	-1.12 (-1.66,-0.57)	-1.75 (-2.45,-1.05)	-2.00 (-2.70,-1.29)	-1.026 (-1.54,-0.51)
zML	-0.57 (-1.16,0.022)	-0.37 (-1.12,0.38)	-0.35 (-1.034,0.33)	-0.32 (-0.85,0.21)
EI	168.81 (161.66,175.96)	175.63 (167.92,183.35)	167.83 (159.30,176.36)	172.63 (166.35,178.92)
nMV (ml/kg*m)	1.57 (1.40,1.73)	1.40 (1.22,1.58)	1.50 (1.34,1.66)	1.55 (1.40,1.71)
nCSA (mm^2/kg)	19.50 (17.66,21.34)	17.65 (15.64,19.67)	17.00 (14.98,19.019)	19.47 (17.75,21.20)
nML (mm/m)	122.59 (116.26,128.93)	124.92 (116.88,132.96)	125.74 (118.71,132.77)	126.72 (121.25,132.19)
GMFCS II+III (n=15)				
zMV	-1.97 (-2.42,-1.52)	-2.33 (-2.90,-1.77)	-2.52 (-3.00,-2.04)	-2.47 (-2.97,-1.98)
zCSA	-1.77 (-2.47,-1.089)	-2.32 (-3.086,-1.56)	-2.53 (-3.27,-1.80)	-2.10 (-2.91,-1.28)
zML	-1.47 (-1.87,-1.089)	-1.34 (-1.87,-0.83)	-1.76 (-2.056,-1.47)	-1.62 (-1.94,-1.30)
EI	163.27 (156.57-169.97)	174.83 (166.05-183.61)	162.20 (156.69-168.51)	167.63 (161.18,174.08)
nMV (ml/kg*m)	1.28 (1.15,1.41)	1.18 (1.00,1.36)	1.14 (0.99,1.29)	1.15 (1.00,1.31)
nCSA (mm^2/kg)	17.24 (15.53,18.95)	15.74 (13.91,17.58)	15.16 (13.45,16.87)	16.23 (14.24,18.22)
nML (mm/m)	112.51 (107.51,117.52)	114.23 (108.12,120.33)	110.81 (106.63,114.98)	112.46 (108.14,116.78)

Estimates and confidence intervals (CI, lower bound, upper bound) at the 4 different timepoints are displayed per GMFCS group. Data are presented as deficits (z-scores) and as normalized outcomes. BL, baseline; 3M, 3 months; 6M, 6 months; 1Y, 1 year; CI, confidence interval; GMFCS, Gross Motor Function Classification System; zMV, z-scores of muscle volume; zCSA, z-scores of cross-sectional area; zML, z-scores of muscle length; EI, echo-intensity; nMV, normalized muscle volume; ml, mililiter; kg, kilogram; m, meter; nCSA, normalized cross-sectional area; mm^2, squared milimeter; nML, normalized muscle length; mm, milimeter

Table S5. Results of the linear mixed model analyses of the normalized muscle size parameters of the GMFCS groups

	nMV (ml/kg*m)			nCSA (mm^2/kg)			nML (mm/m)		
Time*group effect	F-statistics	p-value	$\eta^2 p$	F-statistics	p-value	$\eta^2 p$	F-statistics	p-value	$\eta^2 p$
	1.78	0.16	0.082	0.77	0.52	0.037	0.86	0.47	0.097
Differences	MD/%	p-value		MD/%	p-value		MD/%	p-value	
GMFCS I (n=11)									
3 months – baseline	-0.17/-11%	0.048		-1.85/-9%	0.064		2.33/2%	0.28	
6 months – baseline	-0.07/-4%	0.20		-2.50/-12%	**0.0027**		3.15/+3%	0.27	
1 year – baseline	-0.02/-1%	0.71		-0.03/0%	0.95		4.13/+3%	0.082	

6 months – 3 months	0.10/+7%	0.076	-0.65/-4%	0.48	0.82/+1%	0.65
1 year – 3 months	0.15/+11%	0.064	1.82/+10%	0.091	1.78/+1%	0.38
1 year – 6 months	0.05/+3%	0.23	2.47/+15%	**0.0028**	0.98/+1%	0.42
GMFCS II+III (n=15)						
3 months – baseline	-0.10/8%	0.054	-1.50/-9%	0.088	1.72/+2%	0.29
6 months – baseline	-0.14/-11%	**0.011**	-2.08/-12%	**0.011**	-1.70/2%	0.43
1 year – baseline	-0.13/-10%	0.013	-1.01/-6%	0.22	-0.05/0%	0.98
6 months – 3 months	-0.04/-3%	0.55	-0.58/-4%	0.17	-3.42/-3%	0.29
1 year – 3 months	-0.03/-3%	0.66	0.49/+3%	0.37	-1.77/-2%	0.47
1 year – 6 months	0.01/+1%	0.76	1.07/+7%	0.12	1.65/+1%	0.26

Overview of the timexgroup effect and pairwise mean differences between the different timepoints for the GMFCS groups. For the pairwise mean differences, significant results at the 0.013 alpha-level were indicated in bold. Asterisks (*) indicate significant results at the 0.002 corrected alpha-level for 6 pairwise comparisons. Mean differences of the estimates were calculated as: outcome at later timepoint – outcome at earlier timepoint. Positive mean difference (MD) values indicate an increase of the muscle outcome at the follow-up measurement. nMV, normalized muscle volume; ml, mililiter; kg, kilogram; m, meter; nCSA, normalized cross-sectional area; mm2, squared milimeter; nML, normalized muscle length; mm, milimeter; η^2p, partial eta squared; GMFCS, Gross Motor Function Classification Level; MD, mean difference

Table S6. Comparison between the baseline patient characteristics of the botulinum neurotoxin type A (BoNT-A) naive group and BoNT-A history group

	BoNT-A naive	BoNT-A history	p-value
Age (years)	4.53; 2.09	6.41; 3.24	**0.025**
Body length (m)	1.00; 0.18	1.12; 0.18	**0.016**
Body weight (kg)	15.25; 4.65	18.90; 9.2	**0.008**
zMV	-1.34; 1.09	-1.95; 1.22	0.080
zCSA	-1.04; 1.70	-2.14; 1.59	0.12
zML	-0.91; 1.58	-1.39; 0.65	0.21
EI	160.90; 10.55	170.18; 15.66	0.23
nMV (ml/kg*m)	1.47; 0.28	1.36; 0.37	0.27
nCSA (mm^2/kg)	19.066; 4.84	15.25; 3.64	0.049
nML (mm/m)	116.12; 19.93	116.33; 7.18	0.77

Data are presented as median; IQR (interquartile range). Mann-Whitney U test with significant alpha level 0.05 was applied for anthropometric parameters and were indicated in bold. Bonferroni correction was applied for 4 muscle outcomes (alpha level 0.013). M, meter; kg, kilogram; zMV, z-scores of muscle volume; zCSA, z-scores of cross-sectional area; zML, z-scores of muscle length; EI, echo-intensity; nMV, normalized muscle volume; ml, mililiter; kg, kilogram; m, meter; nCSA, normalized cross-sectional area; mm^2, squared milimeter; nML, normalized muscle length; mm, milimeter

Table S7. Overview of the muscle parameters at the 4 different timepoints per BoNT-A group

	BL	3M	6M	1Y
	Estimate (CI)	Estimate (CI)	Estimate (CI)	Estimate (CI)
BoNT-A naive (n=17)				
zMV	-1.37 (-1.89,-0.86)	-2.023 (-2.62,-1.42)	-2.010 (-2.52, -1.49)	-1.86 (-2.44,-1.27)
zCSA	-1.30 (-2.45,-0.85)	-1.96 (-0.076,0.78)	-1.98 (-0.93,0.32)	-1.65 (-0.84,0.17)

zML	-0.99 (-1.48,-0.49)	-0.82 (-1.44,-0.21)	-1.092 (-1.69,-0.49)	-0.96 (-1.48,-0.45)
EI	164.69 (157.99,171.39)	174.78 (166.63,182.93)	162.14 (155.85,168.42)	170.39 (164.16,176.62)
nMV (ml/kg*m)	1.45 (1.30,1.61)	1.27 (1.083,1.47)	1.30 (1.14,1.46)	1.34 (1.16,1.52)
nCSA (mm^2/kg)	19.00 (17.22,20.80)	17.04 (15.18,18.90)	16.97 (15.32,18.60)	17.89 (15.92,19.86)
nML (mm/m)	117.00 (111.27,122.72)	120.21 (111.37,129.05)	113.65 (106.56,120.73)	118.71 (113.02,124.40)
BoNT-A history (n=9)				
zMV	-2.04 (-2.55,-1.55)	-2.27 (-2.87,-1.68)	-2.061 (2.78,-1.34)	-2.12 (-2.73, -1.51)
zCSA	-1.88 (-2.37,-1.38)	-2.32 (-3.22,-1.41)	-2.86 (-3.70,-2.026)	-1.63 (-2.36,-0.90)
zML	-1.29 (-1.85,-0.74)	-1.36 (-2.084,-0.64)	-0.94 (-1.50,-0.39)	-1.27 (-1.85,-0.69)
EI	167.36 (160.45,174.26)	175.76 (168.17,183.34)	171.60 (165.70,177.50)	168.53 (162.020,175.04)
nMV (ml/kg*m)	1.31 (1.16,1.46)	1.24 (1.059,1.42)	1.30 (1.082,1.52)	1.28 (1.090,1.47)
nCSA (mm^2/kg)	16.67 (15.40,17.94)	15.58 (13.42,17.74)	14.13 (12.00,16.26)	17.07 (14.88,19.24)
nML (mm/m)	116.37 (111.04,121.70)	115.32 (107.18,121.70)	120.31 (113.58,127.04)	118.08 (112.22,123.94)

Estimates and confidence intervals (CI, lower bound, upper bound) at the 4 different timepoints are displayed per BoNT-A subgroup. Data are presented as deficits (z-scores) and as normalized outcomes. BL, baseline; 3M, 3 months; 6M, 6 months; 1Y, 1 year; CI, confidence interval; BoNT-A, Botulinum neurotoxin type A; zMV, z-scores of muscle volume; zCSA, z-scores of cross-sectional area; zML, z-scores of muscle length; EI, echo-intensity; nMV, normalized muscle volume; ml, mililiter; kg, kilogram; m, meter; nCSA, normalized cross-sectional area; mm^2, squared milimeter; nML, normalized muscle length; mm, milimeter

Table S8. Results of the linear mixed model analyses of the normalized muscle size parameters of the BoNT-A groups

	nMV (ml/kg*m)			nCSA (mm^2/kg)			nML (mm/m)		
Time effect*group effect	F-statistics	p-value	η^2p	F-statistics	p-value	η^2p	F-statistics	p-value	η^2p
	0.92	0.44	0.044	2.05	0.12	0.093	3.40	0.034	0.30
Differences	MD/%	p-value		MD/%	p-value		MD/%	p-value	
BoNT- naive (n=17)									
3 months – baseline	-0.18/-12%	**0.0062**		-1.96/-10%	0.023		3.21/+3%	0.16	
6 months – baseline	-0.15/-10%	**<0.0001***		-2.03/-10%	**0.0002***		-3.35/-3%	0.22	
1 year – baseline	-0.11/-8%	**0.0086**		-1.11/-6%	0.050		1.71/+1%	0.38	
6 months – 3 months	0.03/+3%	0.72		-0.07/0%	0.92		-6.56/-5%	0.065	
1 year – 3 months	0.07/+6%	0.36		0.85/+5%	0.33		-1.50/-1%	0.62	
1 year – 6 months	0.04/+3%	0.23		0.92/+5%	**0.15**		5.06/+4%	**0.010**	
BoNT-A history (n=9)									
3 months – baseline	-0.07/-.5%	0.41		-1.09/-7%	0.34		-1.05/-1%	0.64	
6 months – baseline	-0.01/-1%	0.91		-2.54/-15%	0.045		3.94/+3%	0.25	
1 year – baseline	-0.03/-2%	0.66		0.40/+2%	0.70		1.71/+1%	0.48	
6 months – 3 months	0.06/+5%	0.29		-1.45/-9%	0.020		4.99/+4%	0.13	
1 year – 3 months	0.04/+3%	0.48		1.49/+10%	0.038		2.76/+2%	0.23	
1 year – 6 months	-0.02/-2%	0.65		2.94/+20%	**0.0003***		-2.23/-2%	0.13	

Overview of the timexgroup effect and pairwise mean differences between the different timepoints for the BoNT-A groups. For the pairwise mean differences, significant results at the 0.013 alpha-level were indicated in bold. Asterisks (*) indicate significant results at the 0.002 corrected alpha-level for 6 pairwise comparisons. Mean differences of the estimates were calculated as: outcome at later timepoint – outcome at earlier timepoint. Positive mean difference (MD) values indicate an

increase of the muscle outcome at the follow-up measurement. nMV, normalized muscle volume; ml, mililiter; kg, kilogram; m, meter; nCSA, normalized cross-sectional area; mm2, squared milimeter; nML, normalized muscle length; mm, milimeter; η^2p, partial eta squared; BoNT-A, Botulinum neurotoxin type A; MD, mean difference

Table S9. Comparison between the baseline patient characteristics and muscle parameters of the intervention group and control group

	Intervention group	Control group	p-value
Age (years)	5.19 (1.81)	4.98 (2.15)	0.51
Body length (m)	1.054 (0.13)	1.056 (0.15)	0.95
Body weight (kg)	17.97 (5.70)	18.11 (5.35)	0.90
zMV	-1.61 (1.02)	-1.83 (1.22)	0.46
zCSA	-1.50 (1.22)	-1.61 (1.58)	0.27
zML	-1.09 (0.92)	-1.013 (1.16)	0.79
EI	165.61 (12.81)	158.86 (13.81)	0.11
Growth-rate (ml/mo)	0.44 (0.12)	0.44 (0.13)	0.92
nMV (ml/kg*m)	1.40 (0.30)	1.31 (0.31)	0.28
nCSA (mm^2/kg)	18.20 (3.40)	18.026 (4.67)	0.88
nML (mm/m)	116.78 (10.67)	117.069 (12.57)	0.86

Data are presented as mean (standard deviation (SD)). Unpaired t-tests with significant alpha level 0.05 were applied for anthropometric parameters. Bonferroni correction was applied for 4 muscle outcomes (alpha level 0.013). Muscle growth-rate was calculated as ratio of muscle volume (milliliter) per age (months). The growth-rate at follow-up was calculated as the ratio of the change in muscle volume (ml) to the time of follow-up (months). M, meter; kg, kilogram; zMV, z-scores of muscle volume; zCSA, z-scores of cross-sectional area; zML, z-scores of muscle length; EI, echo-intensity; ml, mililiter; mo, month; nMV, normalized muscle volume; ml, mililiter; nCSA, normalized cross-sectional area; mm^2, squared milimeter; nML, normalized muscle length; mm, milimeter

Table S10. Results of the repeated measures ANOVA for the intervention and control group

	Time-effect			Time*group effect		
	F-statistics	p-value	η^2p	F-statistics	p-value	η^2p
nMV (ml/kg*m)	0.22	0.64	0.0043	6.72	**0.012**	0.12
nCSA (mm^2/kg)	0.48	0.49	0.010	0.73	0.40	0.014
nML (mm/m)	2.008	0.16	0.039	0.004	0.95	0.00008

Pairwise comparison

	Intervention group				Control group			
	Baseline	Follow-up	MD/%	p-value	Baseline	Follow-up	MD/%	p-value
nMV (ml/kg*m)	1.40 (1.28,1.52)	1.32 (1.17,1.47)	-0.08 /-6%	0.035	1.31 (1.19,1.43)	1.37 (1.22,1.51)	0.06 /+5%	0.14
nCSA (mm^2/kg)	18.20 (16.60,19.81)	17.60 (15.73,19.47)	-0.60 /-3%	0.28	18.03 (16.42,19.63)	18.09 (16.22,19.96)	0.06 /0%	0.91
nML (mm/m)	116.78 (112.19,121.37)	118.49 (113.93,123.061)	1.71 /+1%	0.30	117.36 (112.77,121.95)	118.93 (114.37,123.50)	-1.57 /-1%	0.34

Overview of the estimates (means) with confidence interval (CI, lower bound, upper bound) for the normalized muscle size parameters per group. Significant results at the 0.013 alpha level were indicated in bold. MD, mean difference; nMV, normalized muscle volume; ml, mililiter; kg, kilogram; m, meter; nCSA, normalized cross-sectional area; mm^2, squared milimeter; nML, normalized muscle length; mm, milimeter

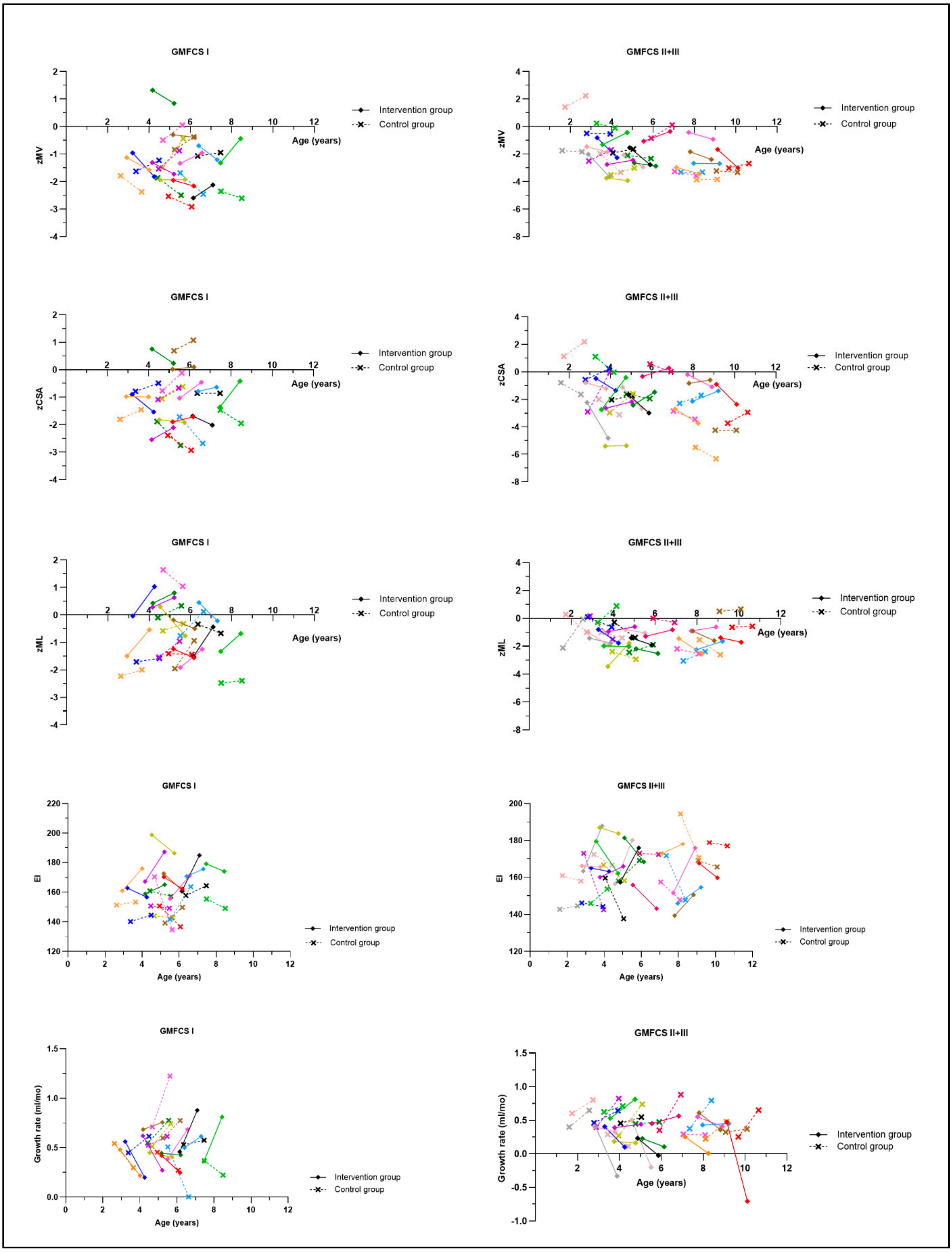
GMFCS I
GMFCS II+III
zMV
Age (years)
Intervention group
Control group
GMFCS I
GMFCS II+III
zCSA
Age (years)
Intervention group
Control group
GMFCS I
GMFCS II+III
zML
Age (years)
Intervention group
Control group
GMFCS I
GMFCS II+III
EI
Age (years)
Intervention group
Control group
GMFCS I
GMFCS II+III
Growth rate (ml/mo)
Age (years)
Intervention group
Control group

Figure S2. Muscle (morphology) data of the intervention group at the baseline and 1 year follow-up measurement (diamond) connected with a full line. Each color represent an induvial child with the matched control (cross, connected with dotted line) in the same color. The graphs are presented per Gross Motor Function Classification Level (I and II+III).
